# Membrane-Enabled Dialysate Regeneration for Wearable and Miniaturized Artificial Kidney Systems: Transport Requirements, Hybrid Architectures, and Translational Constraints

**DOI:** 10.3390/membranes16100311

**Published:** 2026-09-24

**Authors:** Yiqin Gan, Jingying Zhou, Chiwa Ao-Ieong, Shouping Zhu, Yihan Wang, Chen Yun, Xiaofan He, Lianghong Yin, Wenjie Ye, Suhua Xu, Xianggeng Chi

**Affiliations:** 1 Institute of Nephrology and Blood Purification, Jinan University, Guangzhou 510630, China; 2 Department of Nephrology, Red Cross Hospital of Wuzhou, Wuzhou 543000, China; 3 Department of Nephrology, Kiang Wu Hospital, Macao SAR, China; 4 Guangzhou Institute of Technology, Xidian University, Guangzhou 510555, China; 5 Charité–Universitätsmedizin Berlin, Campus Charité Mitte, 10117 Berlin, Germany; 6 Guangdong Medical Devices Quality Surveillance and Test Institute, Guangzhou 510663, China; 7 Guangzhou Enttxs Medical Products Co., Ltd., Guangzhou 510663, China; 8 Department of Nephrology and Rheumatology, The Fifth Affiliated Hospital of Jinan University, Heyuan 517600, China; 9 Department of Nephrology, Xiaolan Clinical Institute, Shantou University Medical College, Zhongshan 528415, China

**Keywords:** dialysate regeneration, membrane separation, nanofiltration, forward osmosis, mixed-matrix membrane, wearable artificial kidney, urea removal

## Abstract

Low-volume dialysate recirculation couples the primary dialysis membrane to the regeneration circuit in wearable and miniaturized artificial kidneys. This narrative review examines membranes as blood–dialysate barriers, fractionation devices, adsorptive supports, and catalyst-containment interfaces. Nanofiltration, forward osmosis (FO), mixed-matrix adsorption, and membrane-supported catalysis are compared using transport, capacity, safety, and operating duration. Among the seven representative regeneration studies tabulated here, five tested only model or synthetic feeds, one operated at 70 °C, and three projected clinical-scale throughput or module requirements from bench data; and one demonstrated 24 h of coupled FO–oxidation bench operation. These overlapping counts describe the selected studies, not the entire literature. Commercial nanofiltration can separate urea from glucose and selected ions, while forward-osmosis and mixed-matrix systems enable urea transfer or capture. Sustained regeneration, nevertheless, requires a destination for the transferred urea: capture in a replaceable sorbent, chemical conversion with product control, or discharge. Membrane selection must, therefore, be evaluated together with electrolyte balance, fouling, return-stream safety, and cartridge capacity. Future studies should combine comparable reporting conditions with complete mass balances, patient-derived spent dialysate, prolonged closed-loop operation, and measured module mass and energy demand.

## 1. Introduction

Conventional hemodialysis sustains life by transferring retained solutes and excess water across a semipermeable membrane, but the treatment remains tied to large volumes of purified water, fixed infrastructure, and intermittent clinical sessions [1]. Portable and wearable artificial kidney systems seek to extend treatment beyond this setting, yet miniaturization changes the separation problem itself. Once the abundant single-pass dialysate supply is replaced by a small recirculating pool, the device must continuously restore the dialysate-side driving force while controlling fluid and electrolyte composition [2,3,4].

The amount of urea that must be removed each day places a substantial demand on the capacity of a compact regeneration cartridge. Urea is small, neutral, highly water soluble, and readily crosses dialysis membranes but binds weakly to many conventional sorbents. Without conversion, capture, or discharge, it accumulates in the recirculating dialysate and reduces the concentration gradient that drives its transfer from blood. Urea management is, therefore, a major obstacle to reducing cartridge mass while maintaining treatment over many hours [5,6,7,8].

Within the primary hemodialyzer, the semipermeable membrane forms the blood–dialysate interface. Its chemistry, pore structure, area, hydraulic permeability, and interactions with proteins determine which solutes cross into the dialysate and how quickly they do so [9,10,11,12,13]. A secondary membrane operates within the dialysate-regeneration circuit and treats the fluid intended for reuse. Depending on its design, it can separate solute streams, support an adsorbent, or isolate a reaction compartment from the fluid intended for reuse.

Separation across a membrane must be distinguished from removal of a solute from the reusable fluid. Transfer of urea to a second liquid compartment does not, by itself, reduce the total amount of urea in the connected circuit. Sustained removal requires the transferred urea to be captured outside the reusable liquid, chemically converted with appropriate product control, or discharged. For example, forward-osmosis water recovery redistributes water and urea between feed and draw solutions [14], whereas a coupled forward-osmosis–photoelectrochemical system also provides an oxidation step that consumes urea [15]. The first process recovers water; the second combines membrane transport with a urea-conversion process. Their performance must be assessed against these different functions.

Broad artificial-kidney reviews provide the context for this subsystem analysis [16]. Ramada et al. discuss dialysate regeneration alongside advanced blood-contacting membranes, bioartificial kidneys, and portable, wearable, and implantable devices [2]. Wieringa et al. examine routes toward greater treatment mobility, including peritoneal dialysis, hemodialysis, nanoporous filters, bioreactors, and oral sorbents [4]. Both already address technologies relevant to regeneration.

The present review concentrates on the recirculating hemodialysis dialysate circuit. It connects the primary membrane that transfers solutes from blood, the secondary membranes that separate or contain process streams, and the adsorption, reaction, or discharge steps that sustain removal. Its contribution is a comparison of these functions using reported operating conditions and quantitative endpoints, including the 2025 commercial-nanofiltration and patient-derived spent-dialysate studies [17,18]. The analysis then identifies the mass-balance, safety, fouling, duration, and integration evidence needed to move from a membrane experiment to a working regeneration module.

### Literature Search and Evidence Selection

This narrative review combines previously identified sources with a documented PubMed/MEDLINE search performed on 4 September 2026. No lower publication-date limit was applied; retrieved records spanned 1971–2026. The first title/abstract query combined “dialysate regeneration” or “dialysis solution regeneration” with membrane*, nanofiltration, “forward osmosis”, “mixed matrix”, adsorp*, urease, electrooxidation, photooxidation, or sorbent*. The second combined “wearable artificial kidney” or “portable artificial kidney” with dialysate or regeneration, and membrane*, sorbent*, or urea. The searches returned 44 and 43 records; removing 16 duplicates left 71 unique records for title and abstract assessment.

Selection focused on hemodialysis dialysate regeneration, portable or wearable hemodialysis, membrane fractionation, urea adsorption or conversion, and membrane-transport or safety evidence needed to interpret these technologies. Human, animal, laboratory, computational, and focused-review publications were considered. Peritoneal-dialysis-only studies, ultrafiltrate regeneration without a hemodialysis dialysate loop, unrelated filtration applications, and reports with only incidental relevance were outside the scope. Original studies with identifiable materials, feeds, operating conditions, transport or adsorption measurements, and duration were prioritized for the quantitative comparison.

The final synthesis cites 50 publications. Bibliographic reconciliation identified 16 of these among the 71 PubMed records and 34 from the existing source set and supplementary reference-list or targeted citation searches. The other 55 PubMed records were not cited in the final synthesis; this number includes relevant but redundant or tangential records and is not an exclusion count for methodological quality. These numbers document the sources used in a narrative review rather than a prospectively screened systematic-review cohort. No meta-analysis was performed, and numerical results were compared within the conditions of the original experiments. ChatGPT (OpenAI; Work mode; GPT-6 Astra Pro for final proofreading) assisted literature searches, extraction and checking of reported data, and drafting of the comparative framework. Yiqin Gan checked the cited evidence and all AI-assisted outputs; Acknowledgments provides the full disclosure.

## 2. Membrane-Centered Architecture of Low-Volume Dialysate Recirculation

### 2.1. Coupled Blood-to-Dialysate Transport

The primary membrane establishes the first transport step from blood to dialysate (Figure 1A). For a freely diffusible solute under diffusion-dominated conditions, a simplified Fick-type relation expresses local solute flux as an overall mass-transfer coefficient multiplied by the concentration difference across the blood–dialysate interface [9,10]:

(1) $$J_{s}=K_{o}(C_{b}-C_{d})$$

The flux, $J_{s}$, measures the amount of solute crossing the unit membrane area per unit time, for example, in mol m^−2^ s^−1^. The coefficient, $K_{o}$ (m s^−1^), combines resistance from the blood-side boundary layer, the membrane, and the dialysate-side boundary layer. The concentrations $C_{b}$ and $C_{d}$ (mol m^−3^) refer to the local blood and dialysate, respectively. The subscripts s, o, b, and d identify solute, overall, blood, and dialysate. A positive concentration difference, $C_{b}$ − $C_{d}$, therefore, produces a positive blood-to-dialysate flux. This local relation describes passive diffusive transport; it does not include convective transport or binding kinetics explicitly.

Urea illustrates why regeneration capacity limits blood-side clearance. Lowering the urea concentration in the recirculating dialysate maintains the driving concentration difference. If urea accumulates in that pool, a larger or more permeable primary membrane cannot fully compensate for inadequate regeneration. Creatinine and other small water-soluble solutes follow the same transport principle, although their production rates and sorbent affinities differ [5,9,10].

Changes to the primary membrane can alter the load that the regeneration circuit must handle. More open pores may allow for additional middle molecules to enter the dialysate. Adsorptive blood-side membranes can also remove protein-bound toxins, but a toxin captured in the membrane is not necessarily transferred to the dialysate. The evaluation must distinguish transmembrane transfer from adsorption within the primary membrane before assigning a downstream treatment load [9,12,13,19].

### 2.2. Secondary Membranes as Regenerative Interfaces

Secondary membranes normally contact dialysate rather than blood. Primary blood-contacting membranes require assessment of hemolysis, coagulation, platelet and leukocyte activation, and complement activation, in addition to protein interactions. For secondary membranes, the principal exposure route is through contaminants that may enter the returning dialysate and cross the primary membrane. Low extractables, chemical stability, microbial control, and membrane integrity are, therefore, essential even when the secondary membrane has no direct blood contact [9,12,13,19].

Figure 1B shows three possible functions of a secondary membrane. Nanofiltration (NF) or forward osmosis (FO) can preferentially transfer urea relative to glucose or selected ions; a mixed-matrix membrane can capture solutes in embedded adsorbent particles; and a membrane around a catalytic compartment can control transport to the reaction zone and help contain the catalyst. The membrane does not always have to contain the solute sink. Adsorption provides a sink within a mixed-matrix membrane, whereas fractionation usually requires a separate capture, conversion, or discharge step. The three rows represent alternative process designs. In any selected design, the complete spent-dialysate flow or a controlled side stream may be treated. Fluid intended for return requires a composition check; a separated treatment stream must be processed or discharged rather than simply rejoined to the pool. The flow arrangement, sink capacity, and treatment of both product streams must be stated for the particular module [15,17,18,20,21,22,23].

### 2.3. Closed-Loop Mass Balance

A conservation-of-mass balance makes the role of regeneration explicit. For one target solute in a well-mixed dialysate pool, the rate of accumulation equals the rate entering across the primary dialyzer minus the rates captured or converted outside the reusable liquid and lost through discharge. Assuming no other external source of that solute, the instantaneous balance is [5,6,15]

(2) $$\frac{d(C_{d}V_{d})}{dt}={\dot{N}}_{dialyzer}-{\dot{N}}_{sink}-{\dot{N}}_{discharge}$$

The product, $C_{d}$ $V_{d}$, is the amount of the target solute in the liquid pool: $C_{d}$ is its concentration (mol m^−3^), $V_{d}$ is the pool volume (m^3^), and t is time (s). Each dotted $\dot{N}$ denotes an amount rate (mol s^−1^). ${\dot{N}}_{dialyzer}$ describes entry from blood; ${\dot{N}}_{sink}$ describes net capture in a retained adsorbent or chemical conversion; and ${\dot{N}}_{discharge}$ describes removal in discarded or sampled fluid. Transfer between connected liquid compartments is an internal flow, not a sink. If those compartments have different concentrations, their individual concentration–volume products must be summed to obtain the total liquid inventory. Integrating the rate balance over a treatment interval results in

(3) $$C_{d}(t)V_{d}(t)-C_{d}(t_{0})V_{d}(t_{0})=\int_{t_{0}}^{t}[{\dot{N}}_{dialyzer}(\tau )-{\dot{N}}_{sink}(\tau )-{\dot{N}}_{discharge}(\tau )]\;d\tau$$

The left side now measures the change in solute amount between the initial time, $t_{0}$, and the later time, t. The integration variable, τ, runs over that interval, so the right side adds the net input and removal rates over time. With a constant pool volume, the inventory change simplifies to $V_{d}$[$C_{d}$(t) − $C_{d}$ $\left(t_{0})\right]$. Adsorption acts as a sink only while the solute remains retained over the treatment interval; exhaustion or desorption reduces its net contribution. Chemical conversion removes the target species but does not necessarily remove its constituent elements: urease consumes urea while leaving nitrogen in ammonia/ammonium, which requires a separate product balance and capture step [24,25,26].

Figure 1 connects blood-to-dialysate transport (A), secondary treatment options and their sinks (B), and the dialysate inventory balance (C). Its bottom row identifies performance measures to apply to the complete system, including flux, selectivity, transmembrane pressure, fouling, safety, and operating duration.

## 3. Membrane Performance Metrics for Device-Relevant Evaluation

### 3.1. Transport and Selectivity

Membrane studies relevant to artificial kidneys should report more than percentage removal. The membrane material, selective-layer structure, configuration, active area, nominal molecular-weight cutoff or pore descriptor, feed composition, temperature, flow orientation, and driving force are needed to interpret transport. Without these variables, performance cannot be scaled from a coupon experiment to a compact module [9,10,11,12,13].

Water transport and solute separation answer different design questions. For a pressure-driven membrane, a simplified hydraulic relation describes water flux as proportional to the hydrostatic pressure difference after allowing for the opposing osmotic pressure [9,10]:

(4) $$J_{v}=L_{p}(\Delta P-\Delta \pi )$$

Water flux, $J_{v}$, is the volume passing through unit membrane area per unit time (L m^−2^ h^−1^). Hydraulic permeance, $L_{p}$ (L m^−2^ h^−1^ bar^−1^), relates that flux to the net driving pressure. The transmembrane hydrostatic and osmotic pressure differences are ΔP and Δπ, respectively, expressed in the same pressure units. With the chosen sign convention, water flows in the positive direction when ΔP exceeds Δπ. This ideal expression assumes that the osmotic pressure difference is fully effective. For membranes that transmit solutes, effective osmotic opposition depends on solute retention; concentration polarization and fouling further affect the observed flux [9,10,17].

Solute rejection describes a different measurement: the fraction by which permeate concentration is lower than feed concentration. The observed rejection of solute i is calculated as [17]

(5) $$R_{i}=1-\frac{C_{p,i}}{C_{f,i}}$$

The concentrations $C_{p,i}$ and $C_{f,i}$ refer to solute i in the permeate and feed, measured in matching units. $R_{i}$ is dimensionless; multiplying it by 100 results in percentage rejection, and 1 − $R_{i}$ produces transmission. High urea rejection is not always the objective. A membrane feeding a urea-treatment side loop may, instead, require high urea transmission relative to glucose and selected electrolytes, together with a measured recovery of the returning fluid [15,17].

Osmotically driven systems require additional reporting of draw composition, reverse solute flux, dilution, water recovery, and the mass-transfer coefficient for the target solute. Mixed-matrix membrane adsorbers need both permeance and dynamic uptake because the permeable scaffold and embedded sorbent contribute different functions. Equilibrium adsorption capacity alone cannot establish runtime [14,15,18,20,21,22,27].

Direct numerical comparison across studies remains limited because neither the test matrix nor the reporting conventions are standardized. Water transport is variously reported as flux, hydraulic permeability or permeance, or total water recovery, whereas solute behavior is reported as rejection, transmission, finite-batch removal, adsorption capacity, a mass-transfer coefficient, or a reaction rate. These quantities describe different processes and are not interchangeable. Their values depend on feed composition and concentration, temperature, pH, hydrostatic or osmotic driving force, cross-flow or recirculation hydrodynamics, active area, runtime, and sampling method. In a regeneration circuit, apparent performance also reflects coupled concentration polarization, fouling, adsorbent saturation, reaction kinetics, draw-solution dilution, and reverse solute flux. Permeance, rejection, or selectivity alone, therefore, does not capture the full multiphysics or predict integrated closed-loop performance. A common benchmark should use a physiological multisolute feed at 37 °C, defined hydrodynamics and solute loading, active-area normalization, time-resolved mass balances, fouling or breakthrough data, and post-treatment return-stream quality [14,15,17,18,19,20,21,22,27].

### 3.2. Dynamic Capacity, Fouling, and Integrity

A recirculating device is governed by time-dependent performance. Dynamic capacity should be calculated by integrating the difference between inlet and outlet solute mass-flow rates, with corrections for sampling, discharge, and changes in liquid holdup. Uptake should be normalized to total membrane-module mass or volume, rather than active-powder mass alone. Breakthrough, selectivity drift, pressure increase, and capacity recovery after cleaning or regeneration should be shown over the intended operating interval [18,20,21,22,27].

Fouling must be interpreted according to location. Blood-side membranes face protein adsorption and deposition, cellular interactions, coagulation, platelet and leukocyte activation, and complement activation. Dialysate-side modules avoid most cellular components but still encounter protein traces, precipitates, microbial products, reaction products, and concentration polarization. For mixed-matrix systems, particle loss and polymer swelling are additional failure modes [9,12,13,18,19,20,21,22,27]. Table 1 lists the minimum membrane-specific information needed for device-relevant interpretation.

## 4. Pressure- and Osmotically Driven Membrane Fractionation

### 4.1. Nanofiltration for Small-Solute Fractionation

Nanofiltration separates solutes through the combined effects of molecular size and membrane charge [17,28]. In a regeneration circuit, the objective may be to transmit urea preferentially while retaining glucose or controlling electrolyte distribution. A membrane selected for this task must, therefore, be tested with a representative multisolute feed; a salt-rejection rating obtained for water purification cannot establish its suitability for dialysate.

A 2025 study compared six commercial nanofiltration membranes using urea, glucose, and mono- and divalent ions under variations in pressure, temperature, and feed composition [17]. The lowest tested pressure was 20 psi; there was no below-20-psi baseline. For the healthy model feed at 22 °C, mean glucose rejection at 20 psi ranged from approximately 23% for dnf80 to 84% for NF270. At 40 psi, the approximate mean values were 39%, 78%, 86%, 87%, 73%, and 39% for NF3, NF4, NF90, NF270, dnf40, and dnf80, respectively; at 60 psi, the corresponding values were 37%, 84%, 87%, 80%, 76%, and 41%. These values were read from the reported figure and are, therefore, stated as approximations. Most of the pressure-associated change occurred between 20 and 40 psi and was membrane-dependent rather than universal. Higher temperature modestly increased flux and reduced divalent-ion rejection, while urea rejection was less sensitive to feed variation than glucose rejection.

The pressure- and feed-dependent rejection values support nanofiltration as a fractionation step and show why a manufacturer’s nominal salt rejection is insufficient for module selection. A membrane that transmits urea may also redistribute calcium, magnesium, sodium, potassium, or glucose. Its evaluation requires balances for these solutes and a defined treatment or discharge route for the separated urea stream [17].

Reverse-osmosis membranes provide a tighter solution-diffusion barrier, but neutral urea remains difficult to separate from water without substantial pressure or tailored polymer chemistry. Surface modification of polyamide reverse-osmosis membranes has been investigated specifically to alter urea transport [28]. This mechanistic work expands the membrane-design space, but it was not an integrated test with patient spent dialysate; any regeneration concept would still need to account for pressure, water recovery, electrolyte redistribution, and the fate of both product streams.

### 4.2. Forward Osmosis and Reverse-Osmosis Coupling

Forward osmosis can recover water or transfer urea using an osmotic gradient rather than a large hydraulic pressure. In a spent-dialysate recycling study using a commercial cellulose-triacetate forward-osmosis membrane and dialysis concentrate as the draw solution, approximately 64% water recovery was achieved [14]. Urea rejection remained low because urea is small and uncharged. Flux declined as the feed concentrated and the draw diluted, and visible scaling developed after repeated cycles, although osmotic backwash largely restored flux. The study demonstrates water recovery, not complete dialysate regeneration.

Forward-osmosis membrane orientation, water flux, reverse salt transport, and fouling have also been assessed with actual spent dialysate [27]. Patient-derived fluid makes the fouling evidence more relevant than clean model feeds, yet the system still functions principally as a water-recovery step. Urea transfer and draw-solution management prevent the result from being interpreted as complete toxin removal or as direct proof that the product can be returned safely to a patient-facing loop.

A different forward-osmosis design deliberately used rapid urea transport. Shao and colleagues coupled a commercial aquaporin forward-osmosis cartridge to a protected photoelectrochemical oxidation loop [15]. In forward-osmosis mode, the reported urea mass-transfer coefficient was 1.2 µm s^−1^. At a dialysate flow of 400 mL/min, the membrane stage was projected to support more than 30 g of urea transfer per day, while the coupled oxidation module removed urea at 0.32 mg cm^−2^ h^−1^.

Shao et al. also reported 24 h of coupled FO–oxidation bench operation with human spent dialysate and a continuous urea input [15]. This supports day-long coupling of transport and reaction, although clinical-scale throughput remained extrapolated. The rejection values for this study in Table 2 were obtained in RO-mode characterization; they are not FO operating rejection values. In the FO architecture, reaction fluid recirculates separately rather than returning directly to the primary dialyzer. Wearable validation still requires joint measurements of water and electrolyte balance, energy, product control, and membrane performance at the intended device scale.

## 5. Mixed-Matrix and Adsorptive Membranes

### 5.1. Combining Permeation and Adsorption

Mixed-matrix membrane adsorbers embed a sorptive phase within a porous polymeric support. The support creates a defined flow path and short diffusion distance, while the filler supplies affinity that the polymer lacks. This arrangement can reduce particle handling and improve the accessible surface area, but the measured capacity must be normalized to the complete membrane rather than to filler alone [18,20,21,22].

Cellulose-acetate membrane adsorbers containing activated carbon, ZSM-5, or clinoptilolite were tested with urea, creatinine, and uric acid solutions [20]. Across the formulations, hydraulic permeability was approximately 600–1500 L m^−2^ h^−1^ bar^−1^. At 24 h, batch urea removal was 2% for unfilled cellulose acetate and 16–28% for the filled membranes. These finite-batch removal percentages should be distinguished from the dynamic breakthrough measurements used to estimate cartridge size. Filler retention and electrolyte stability during prolonged clinical use remain to be established.

A hollow-fiber mixed-matrix membrane containing polystyrene-based ninhydrin particles in a polyethersulfone/polyvinylpyrrolidone matrix achieved 3.4 ± 0.3 mmol of urea removal per gram of particles during 4 h of recirculation [21]. Incorporation improved particle accessibility and led to an estimated membrane mass of 215 g for daily urea handling. The experiment, however, was performed at 70 °C to accelerate covalent binding. That temperature is incompatible with direct clinical recirculation, so the reported capacity cannot be transferred to a wearable device without redesign of kinetics and thermal management.

### 5.2. Dual-Layer Hollow-Fiber Architectures

Dual-layer hollow fibers allow for different functions to be assigned to the lumen and shell sides. A three-component polysulfone/activated-carbon and polysulfone/poly (methyl methacrylate) membrane reached a pure-water flux of 25.2 L m^−2^ h^−1^ and produced a combined average creatinine-plus-urea removal of 29.3% in ultrafiltration–adsorption testing [22]. The result shows that co-adsorption is possible, but percentage removal from a finite test volume remains less informative than dynamic capacity and breakthrough under continuous loading.

More recent work incorporated amine-functionalized mesoporous silica nanoparticles into the outer layer of a dual-layer hollow-fiber mixed-matrix membrane [18]. The optimized membrane showed water permeability of 298.6 ± 3.2 mL m^−2^ h^−1^ mmHg^−1^ and reported adsorption capacities of 523.5 mg/g for urea and 28.1 mg/g for creatinine. In actual patient spent dialysate, reported removals were 74.4% for urea, 56.0% for creatinine, 16.1% for hippuric acid, and 58.7% for spiked lysozyme. Use of real spent dialysate and multisolute assessment are important advances, although wearable-duration breakthrough, module mass, sterilization, and repeated-use data remain necessary.

### 5.3. Distinguishing Blood-Side Mixed-Matrix Membranes

Blood-side mixed-matrix membranes perform a different task from secondary dialysate-regeneration membranes. Geremia et al. tested an activated-carbon/polyethersulfone/polyvinylpyrrolidone fiber for simultaneous removal of protein-bound toxins from human plasma and endotoxin from dialysate [19]. Its water permeance was 11.4 L m^−2^ h^−1^ bar^−1^. In combined 4 h experiments, area-normalized removal reached (415 ± 59) × 10^3^ endotoxin units m^−2^, 2267 ± 159 mg m^−2^ hippuric acid, and 494 ± 96 mg m^−2^ indoxyl sulfate. The proposed 0.15 m^2^ area for daily toxin handling was an extrapolation from these experiments. The study supports a primary blood-purification and contamination-barrier function, not direct removal of the dialysate-side urea load.

Table 2 brings together water transport, urea performance, and co-solute or safety measurements from the representative studies. Numerical ranges cover membranes or conditions tested within a study. NR indicates that a quantitative value was not identified in the source material used for the table. Feed composition, temperature, driving force, normalization, and operating duration are retained alongside the values so that readers can judge which results can be compared [14,15,17,18,19,20,21,22].

For the Abstract counts, the denominator is the seven direct regeneration studies [14,15,17,18,20,21,22]; the blood-side study [19] is excluded. Five tested no patient-derived fluid [14,17,20,21,22], one used 70 °C [21], and three projected daily throughput or cartridge requirements [15,20,21]. One study [15] reported 24 h of coupled FO–oxidation bench operation with human spent dialysate; this was not a clinical wearable trial. Categories overlap and do not estimate proportions in the entire literature.

## 6. Membrane-Coupled Catalytic Urea Conversion

### 6.1. Urease and Downstream Ammonium Capture

The Recirculating Dialysis (REDY) sorbent cartridge established the historical precedent for regenerating a small dialysate volume by converting urea with urease and managing the products with downstream sorbents [24,25,26]. Historical REDY use demonstrated the feasibility of this conversion-and-capture sequence but does not establish the performance of contemporary wearable devices. Later portable and wearable concepts inherited its conversion-capture sequence rather than a complete commercial solution.

Urease hydrolyzes urea to ammonia/ammonium and carbon dioxide. Immobilizing the enzyme on or behind a membrane can retain the catalyst and define the contact area but leaves the nitrogen-containing products to be managed. The regeneration circuit must capture ammonium, control carbon dioxide and pH, restore electrolyte composition, and detect breakthrough before fluid returns to the primary dialyzer. The membrane provides catalytic support or containment; downstream ion exchange retains ammonium until its usable capacity is exhausted [23,24,25,26].

A urease-coated reverse-osmosis membrane has been tested for urea removal in an ultrapure-water system [23]. The study illustrates how a membrane surface can localize conversion and change apparent urea transport, but the simplified feed and the production of ammonia/ammonium and carbon dioxide preclude direct extrapolation to regenerated clinical dialysate. Downstream product capture and a complete electrolyte balance remain essential.

Regenerable potassium- and phosphate-binding sorbent systems have demonstrated relevant multisolute control in vitro and in awake goats [29,30]. These studies support using separate cartridge stages to control different solutes while also showing how downstream conditioning adds to system mass. A membrane-enabled urease module is useful only if catalyst retention or improved mass transfer reduces the total cartridge burden after ammonium capacity and safety margins are included.

### 6.2. Electrochemical and Photoelectrochemical Sinks

Electrochemical oxidation can provide an active sink for a membrane-generated urea side stream. Early reappraisal and miniature-device studies demonstrated urea conversion, including proof-of-principle operation in awake goats [31,32]. More recent nickel–metal hydroxide foams have been investigated as catalytic electrodes for simplified devices [33]. These systems can reduce reliance on enzyme cartridges, but electrode area, current efficiency, power, gas evolution, heat, and durability must be included in the device balance.

Reaction products determine whether urea conversion yields reusable dialysate. Electrooxidation of glucose-containing dialysate generated glucose degradation products under the tested conditions [34]. Depending on electrode chemistry and feed composition, ammonium, nitrite, nitrate, reactive chlorine species, and organic products may also require measurement. Urea disappearance alone is, therefore, insufficient to demonstrate chemical safety [31,32,34,35].

Light-driven and photoelectrocatalytic approaches have also been investigated for urea conversion [36,37]. A TiO_2_-nanowire device removed 14.2 g of urea over 24 h under the reported conditions while monitoring ammonia-, nitrate-, and chlorine-containing products; activated-carbon polishing reduced residual chlorine [35]. The experiment connects daily urea removal with product monitoring, although catalyst stability, light-source durability, and prolonged operation in a complete regeneration circuit remain separate questions.

A selective membrane may improve catalytic regeneration by limiting glucose or other competing solutes entering the reaction compartment and by retaining catalytic material. The FO–photooxidation study provides an experimental example [15]. Whether adding a membrane benefits a portable device depends on the resulting total module mass, volume, pumping energy, reagent consumption, and polishing capacity. Those quantities should be compared with an alternative cartridge designed for the same solute load and duration [15,34,35,36,37].

## 7. Sorbent and Electrolyte Management Within a Membrane-Enabled Circuit

Several cartridge functions remain necessary after membrane separation. Activated carbon captures selected organic solutes and can polish reaction products; zirconium phosphate captures ammonium and exchanges cations; and hydrous zirconium oxide supports phosphate and anion control [24,25,26]. The removal of organic solutes by activated carbon varies with solute identity and treated volume, so its role as a multisolute conditioner should be distinguished from direct urea adsorption [38,39].

Direct urea sorbents attempt to avoid ammonium formation. Candidate classes include MXenes, widened-spacing MoS_2_, reactive ninhydrin- and phenylglyoxal-type polymers, molecularly imprinted particles, and computationally selected porous materials [40,41,42,43,44,45,46,47]. Their reported affinities arise from different mechanisms and test conditions, so equilibrium uptake values should not be compared as though they represented identical cartridge performance.

Molecular imprinting has also been implemented directly in a membrane tested for the transport of urea, creatinine, and vitamin B12 [48]. The diffusion-cell experiment supports a recognition-based membrane concept, but it does not establish selectivity in complete dialysate, dynamic capacity under continuous loading, or a clinically acceptable sink for transmitted urea.

Dynamic capacity and mass remain limiting. In a closed-loop phenylglyoxal column study, model-based estimates under the tested assumptions indicated approximately 0.6 kg of dry sorbent for an 8 h treatment and 1.0 kg for a 2.5 h treatment; the corresponding wet masses were about 2.1 and 3.5 kg, respectively [45]. The authors, therefore, considered the system unsuitable for wearable use and more compatible with portable or bedside operation. Computational screening can prioritize nanoporous candidates, but it cannot establish aqueous stability, packing density, pressure drop, leaching, or manufacturability [49]. A membrane support may improve particle containment and access, yet it also adds polymer mass and transport resistance.

Membranes, sorbents, and reaction modules should, therefore, be selected for complementary tasks. A membrane may direct a smaller treatment stream to a sorbent, embed the adsorbent in a permeable support, or protect a catalytic compartment. Each additional stage also adds volume, flow resistance, or potential failure points. A proposed combination needs to demonstrate a benefit in total cartridge mass, energy use, or return-stream safety under the same treatment conditions.

## 8. Fouling, Integrity, and Long-Duration Operation

Short experiments can conceal the main failure modes of membrane-enabled regeneration. Concentration polarization raises the local solute concentration at a selective layer and can change apparent rejection. Organic deposition or salt precipitation reduces permeance and may shift selectivity. In forward osmosis, draw dilution and reverse solute flux evolve during operation; in adsorptive membranes, surface saturation and pore blockage occur simultaneously [14,18,20,21,22,27].

Dialysate-side fouling is less biologically complex than direct blood contact, but it is not negligible. Spent dialysate can contain low concentrations of proteins, lipid-associated material, microbial fragments, and precipitates. Urease and electrochemical modules can add carbonate, gas, pH shifts, metal ions, reactive products, or fine particles. These species can foul the secondary membrane or alter its surface charge, so tests with clean urea solution represent only an early screening stage [14,15,23,27,34,35].

Integrity testing should match the architecture. Nanofiltration and forward-osmosis modules require pressure or osmotic cycling, defect detection, and solute-leak monitoring. Mixed-matrix membranes require quantitative filler-release and extractables assays. Catalyst-retaining membranes require confirmation that enzymes, nanoparticles, or electrode products cannot reach the return stream. Modules placed at the blood interface also require hemolysis, complement, coagulation, platelet, leukocyte-activation, and protein-adsorption assessment [9,12,13,18,19,20,21,22,27].

Cleaning may recover water flux without restoring adsorption capacity or solute selectivity. Osmotic backwashing recovered flux after FO scaling in one study [14]; reactive urea sorbents pose different challenges because capture can involve chemical binding [42,45]. Reuse claims should, therefore, report capacity, selectivity, integrity, and leaching after complete treatment-and-cleaning cycles.

A convincing durability study should run a full recirculating mass balance at realistic temperature, flow, solute production, and dialysate composition. It should report pressure, flux, rejection, electrolyte drift, by-products, breakthrough, and integrity over time, including the response to capacity exhaustion or membrane failure. A 24 h coupled bench experiment demonstrates sustained module operation; it does not by itself establish ambulatory device performance [3,14,15,18,20,21,22,27,30,32,35].

## 9. Hybrid Architectures, Monitoring, and Translational Readiness

Figure 2 groups three ways to combine membrane transport with sustained solute removal. In the FO–oxidation route, dialysate stays in its own circuit, while a separate reaction-fluid loop receives urea across the membrane and recirculates through oxidation and product polishing. The other routes capture urea within a replaceable mixed-matrix hollow-fiber module or convert it with supported urease before ammonium capture and fluid rebalancing. Each design must identify where the solute is retained or converted and how the returning dialysate is checked.

Monitoring should be matched to the failure mode. Pressure and flow sensors can identify membrane blockage or fiber failure; conductivity and ion-selective measurements can detect electrolyte drift; temperature and gas sensors are important for active reaction modules; and product-specific assays are required for ammonium, reactive chlorine species, nitrate, or organic degradation products. A sensor has clinical value only when it triggers a defined response, such as diverting dialysate, switching a cartridge, reducing current, or stopping treatment [3,24,30,34,35].

A human wearable artificial kidney (WAK) feasibility study demonstrated ambulatory small-solute clearance over a planned 24 h treatment, with five of seven participants completing the full period. The study stopped early following technical problems that included carbon-dioxide bubbles and unstable blood and dialysate flow [3]. Human feasibility evidence for urease-sorbent regeneration, therefore, coexists with unresolved operational problems. The membrane-fractionation, mixed-matrix adsorption, and membrane-coupled oxidation studies discussed here provide primarily laboratory or preclinical evidence.

Wearable extracorporeal hemodialysis, like conventional treatment, requires vascular access for blood withdrawal and return. The 24 h WAK trial used an existing functioning double-lumen tunneled dialysis catheter [3]. It did not test ambulatory use with fistula or graft needles. Movement can increase concerns about needle displacement and bleeding, while catheters introduce infection and thrombosis risks. Access-specific testing, secure connections, pressure and blood-leak monitoring, and automatic clamp or pump-stop responses remain necessary [2,3,4,50].

Table 3 uses five equally weighted evidence domains, each scored from 0 to 2. Evidence level: 0, material or standalone membrane-module testing; 1, a coupled regeneration train or animal testing; 2, human testing. Feed realism: 0, a single-solute model; 1, multisolute or ex vivo patient fluid; and 2, an operating patient blood–dialysate circuit. Flow-operation duration: 0, 4 h or less or unreported; 1, more than 4 h but less than 24 h; 2, at least 24 h. Static adsorption times do not qualify. Safety: 0, separation data alone or no hazard testing; 1, selected cytotoxicity, contaminant-barrier, or operational-failure endpoints; and 2, chemical and biological safety assessment plus fault testing. Safety concerns the return stream, or the blood interface for the primary-membrane comparator. Device integration: 0, a material/coupon; 1, a module or coupled prototype; and 2, reproducible portable/wearable integration with manufacturing evidence. Totals of 0–2, 3–5, 6–8, and 9–10 indicate Low, Low to moderate, Moderate, and High readiness.

## 10. Research Priorities for Membrane-Centered Translation

A complete mass balance should account for feed, retentate, permeate, draw solution, adsorbent, reaction products, and sampled or discharged volumes. A lower feed concentration may result from dilution or transfer to another compartment. Measuring solute amounts in all relevant compartments establishes whether urea was retained, converted, or discharged, which is especially important when NF or FO is designed to transmit urea [14,15,17].

Device projections should report membrane area, complete module mass and volume, active filler mass, energy consumption, and treated solute load together. A projected capacity of 15–30 g urea per day is useful only with the corresponding flow, membrane and electrode area, pump power, draw or reagent use, polishing capacity, and safety margin [15,18,21,35,45].

Feed complexity should increase as a module develops. Buffered urea solutions can support initial screening, followed by complete synthetic dialysate, variations in glucose and ion concentrations, patient-derived spent dialysate, and an integrated dialyzer loop. Commercial-NF screening and the amine-silica hollow-fiber study illustrate the additional information obtained from physiological feed variations and patient fluid [17,18].

The intended function should determine the selectivity endpoint. Urea transfer to a treatment stream calls for transmission relative to glucose and electrolytes; depyrogenation calls for control of endotoxin passage; and water recovery calls for water flux together with urea and salt balances. A single percentage-removal value cannot represent all three functions [14,15,17,18,19,20,21,22,27].

Safety assessment must follow the fluid that will return to the primary dialyzer. Catalytic studies should quantify products, and adsorptive membranes should be tested for particle release and extractables. Tests at saturation, abnormal pressure, or loss of power should establish when to divert fluid or stop treatment. Acceptance limits for the return-stream composition need to be specified before those experiments [15,18,19,20,21,22,23,34,35].

Manufacturing constraints should be considered during material selection. Hollow-fiber spinning, potting, sterilization, shelf stability, variation between batches, and replacement procedures can determine whether laboratory performance survives module production. Membrane optimization should, therefore, use a defined target for module size, lifetime, and maintenance [9,12,13,18,19,20,21,22].

## 11. Conclusions

Low-volume artificial kidney systems require more than a high-performance dialyzer. The blood-side membrane and the dialysate-regeneration circuit form a coupled separation system: the first transfers the toxin load, and the second must provide a sustained sink that preserves the driving force. A secondary membrane can make that sink smaller or safer by fractionating solutes, concentrating urea, retaining a catalyst, or embedding an adsorptive phase.

The selected studies demonstrate complementary capabilities: commercial NF distinguishes small solutes, FO can supply urea to a catalytic compartment, and mixed-matrix hollow fibers provide adsorption under flow or with patient-derived dialysate [15,17,18,21]. One of the seven direct regeneration studies in Table 2 reports 24 h of coupled FO–oxidation bench operation with human spent dialysate [15]. This supports sustained coupling of transport and reaction, but measured wearable performance has not been established for these seven regeneration platforms.

The next experiments should connect those capabilities to explicit device requirements. For NF and FO, a proposed benchmark is closure of water, urea, glucose, and major-electrolyte balances at 37 °C during at least 24 h of dynamic operation with a defined input load and representative spent dialysate. Systems already tested for 24 h should demonstrate repeatability, longer operation, and responses to faults. For adsorptive fibers, breakthrough must be related to total cartridge mass, filler retention, sterilization, and storage. For catalytic systems, nitrogen and carbon products, energy, electrode area, and polishing capacity should be measured together. These are proposed research benchmarks, not established clinical acceptance standards [14,15,17,18,21,34,35].

Progression to animal and human feasibility testing will require stable clearance and fluid control together with a demonstrated response to saturation, leakage, and power loss. Hybrid modules offer a practical route for testing these requirements because transport, capture or conversion, and return-fluid monitoring can be assigned separate functions. The contribution of this review is to make their interactions explicit and to define the evidence needed for integration, complementing the broader device perspectives in References [2,4].

## Figures and Tables

**Figure 1:**
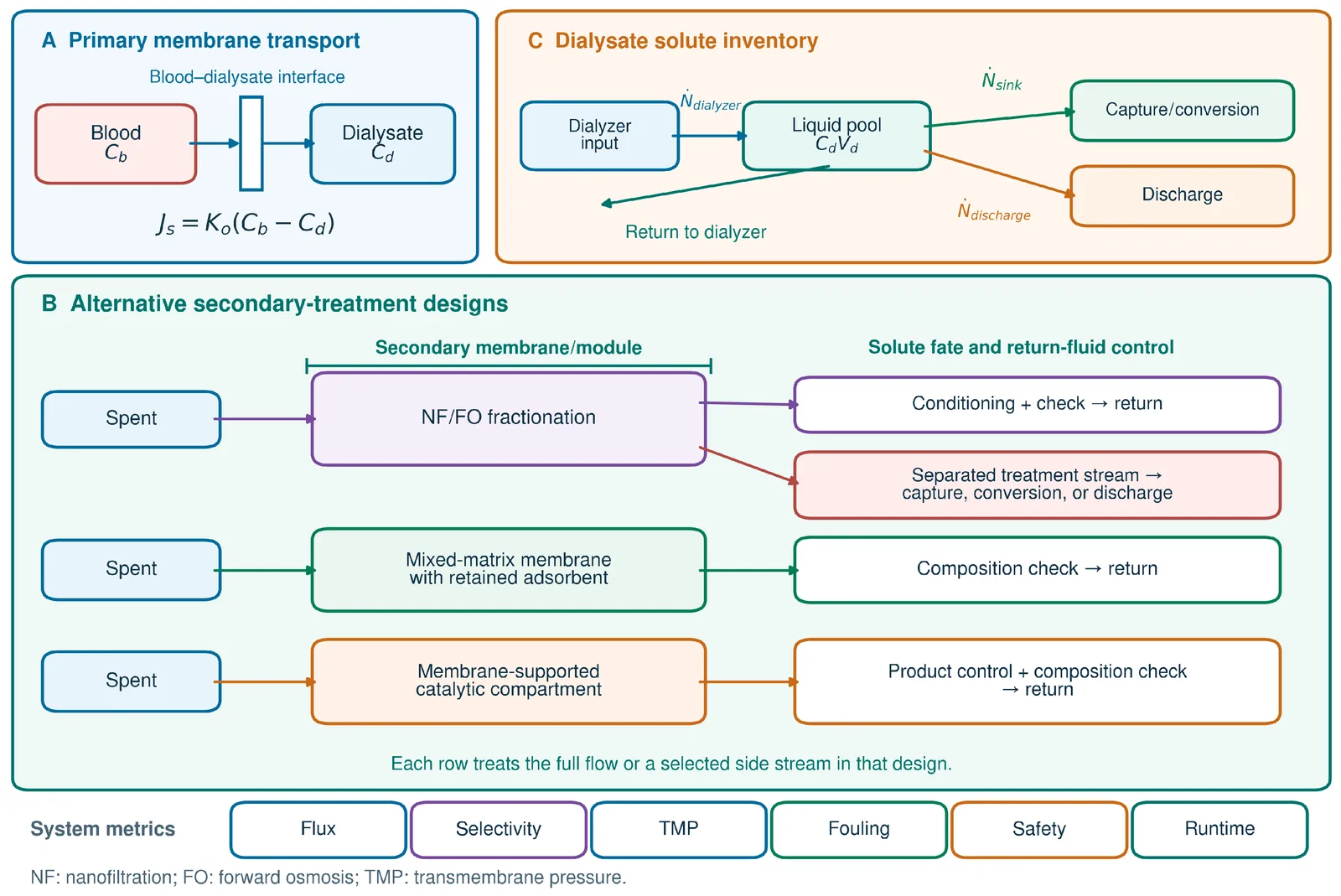
Membrane functions and solute fate in a low-volume dialysate circuit. (**A**) Diffusive transport across the primary blood–dialysate membrane. (**B**) Three alternative secondary-treatment designs, shown in separate rows. “Spent” denotes the full spent-dialysate flow or a selected side stream in that design. NF/FO fractionation requires treatment or discharge of the separated solute stream; the other liquid stream requires composition checking before return. Mixed-matrix adsorption retains solute within the membrane. Membrane-supported catalysis requires product control before return. The bracket covers the membrane/module column only; regenerated dialysate is the downstream product. (**C**) The solute inventory changes according to entry, net capture or conversion, and discharge. Transfer between connected liquid compartments alone does not decrease the total inventory. FO, forward osmosis; NF, nanofiltration; TMP, transmembrane pressure. The bottom row lists system evaluation metrics.

**Figure 2:**
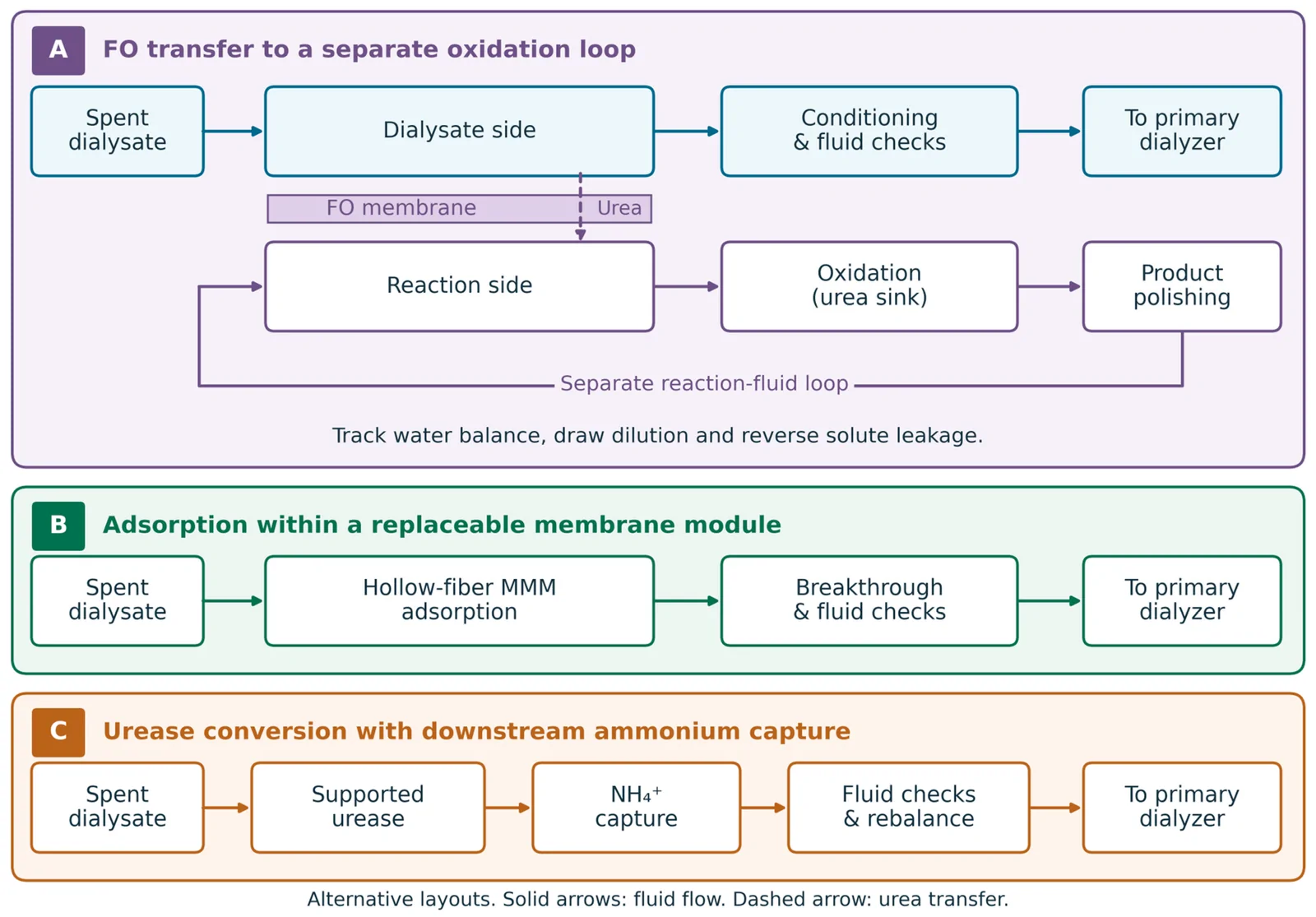
Three alternative hybrid regeneration architectures. (**A**) FO couples separate dialysate and reaction-fluid paths; the dashed arrow denotes urea transfer across the membrane, and reaction fluid returns to the reaction side after oxidation and polishing. The membrane is not an absolute barrier to water or other solutes. (**B**) Hollow-fiber MMM adsorption with breakthrough monitoring. (**C**) Supported urease followed by ammonium capture and fluid rebalancing. These are conceptual alternatives, not a clinically validated circuit or a required flow split. Composition checks precede dialysate return. Abbreviations: FO, forward osmosis; MMM, mixed-matrix membrane; NH_4_^+^, ammonium.

**Table 1:** Minimum reporting framework for membrane-enabled dialysate regeneration.

Domain	Report Explicitly	Reason for Device Relevance
Membrane identity	Polymer or inorganic phase; selective layer; filler; surface modification	Controls transport, stability, leaching, sterilization, and manufacturability
Configuration	Flat sheet, hollow fiber, dual layer, cartridge geometry, active area	Determines packing density, pressure drop, dead volume, and scale-up
Pore or transport descriptor	Pore size, MWCO, permeability, or target-solute mass-transfer coefficient	Allows for comparison of the mechanism and expected solute range
Operating condition	Feed composition, temperature, pH, flow, ΔP or osmotic driving force	Prevents favorable laboratory conditions from being mistaken for clinical performance
Flux and recovery	Water flux or permeance; permeate recovery; reverse solute flux where relevant	Links membrane area to pump, volume, and energy requirements
Solute performance	Feed and product concentrations; rejection or transmission; pairwise selectivity	Shows whether urea handling preserves glucose and electrolyte composition
Dynamic capacity	Breakthrough curve and integrated uptake normalized to full module mass or volume	Determines cartridge size and usable runtime
Fouling and cleaning	Flux decline, selectivity drift, pressure rise, cleaning method, recovery	Defines long-duration stability and reuse
Integrity and safety	Particle release, extractables, catalyst containment, endotoxin barrier, by-products	Protects the returning dialysate and, indirectly, the patient
Integrated runtime	Closed-loop duration, solute loading, mass balance, energy, failure mode	Distinguishes component feasibility from device feasibility

The reporting domains and their relevance to device design were assembled from the membrane-transport and dialysis-engineering principles reviewed in [9,10,11,12]. Table 1 is a practical reporting checklist, not a validated scoring instrument. MWCO, molecular-weight cutoff; ΔP denotes the transmembrane hydrostatic pressure difference. Background shading distinguishes the header and alternating rows only; it does not encode quantitative values or evidence levels.

**Table 2:** Representative membrane platforms for dialysate regeneration or closely coupled artificial-kidney functions.

Platform/Configuration	Feed, Conditions, and Runtime	Water Transport	Urea Performance	Co-Solutes/Safety Endpoints	Interpretive Boundary
Commercial NF screening: six membranes [17]	Model physiological feeds; healthy, hyperglycemic, and hyperkalemic compositions; 20, 40, and 60 psi; mainly 22 °C, with 37 °C tests for dnf40/dnf80; no integrated closed-loop runtime	Permeate flux approximately 0.007–0.42 L m^−2^ h^−1^ across the six membranes and 20–60 psi; this is flux, not pressure-normalized permeance.	Healthy feed: NF3/dnf80 ≤ 10% rejection; NF4/dnf40 approximately 5–20%; NF270 up to 35% with high variability; NF90 up to 57%. NF90 fell below 10% at 60 psi with hyperglycemic/hyperkalemic feeds.	Approximate glucose rejection: 23–84% at 20 psi, 39–87% at 40 psi, and 37–87% at 60 psi. Monovalent-ion rejection was generally <30% except for NF90; divalent-ion rejection was generally >40% except for NF3/dnf80 and was often >70% for NF90/NF270.	No complete regeneration loop or downstream urea sink; pressure, temperature, membrane identity, and feed composition altered the apparent selectivity.
FO water recovery: commercial CTA membrane [14]	Synthetic spent dialysate; dialysis concentrate as draw; repeated cycles; no integrated 24 h run	Approximately 64% water recovery; final partially diluted concentrate volume approximately four fold the initial volume; flux declined and was almost restored by osmotic backwash.	Urea rejection was described as relatively low; quantitative rejection NR.	Phosphate/carbonate scaling became evident after three cycles; quantitative glucose and electrolyte recovery NR.	This process recovers water rather than removing the urea inventory; scaling and draw-stream composition remain limiting.
FO–photoelectrochemical hybrid: commercial aquaporin FO cartridge plus oxidation unit [15]	Model solutions and human spent dialysate; 24 h coupled FO–oxidation bench run with continuous urea input	Excess-water removal 0.081 g cm^−2^ h^−1^, normalized to electrode area.	RO characterization: ≈20% rejection. FO operation: mass-transfer coefficient 1.2 µm s^−1^. Coupled oxidation: 0.32 mg cm^−2^ h^−1^. Projected membrane-stage transfer >30 g day^−1^ at 400 mL min^−1^.	RO characterization: ≈80% rejection of creatinine and ammonium; ≈95–99% for most other tested solutes. These are not FO rejection values.	A 24 h coupled bench result, not clinical wearable validation; daily throughput and required area remain projections, with water/draw balance, power, and product control requiring device-scale assessment.
Cellulose acetate MMMA with AC or zeolite fillers [20]	Single-toxin and mixed aqueous feeds; batch tests to 24 h; dynamic flow 0.5–10 mL min^−1^; no patient-derived dialysate	Hydraulic permeability approximately 600–1500 L m^−2^ h^−1^ bar^−1^ across formulations.	Batch urea removal at 24 h: pristine CA 2%, ZUF 19%, ZSM-5 16%, and AC 28%; urea was the earliest breakthrough solute.	Batch creatinine removal: CA 5%, ZUF 20%, ZSM-5 10%, AC 21%; uric-acid removal by CA was 97%. A combined 12 L cartridge was projected for 90% multisolute removal in 3.5 h.	Batch and dynamic endpoints are not interchangeable; the projected cartridge remains too large for wearability and lacks long-duration validation.
Ninhydrin hollow-fiber MMM: PES/PVP with polystyrene-ninhydrin particles [21]	30 mM urea model dialysate; TMP 0.15 bar; 20 mL min^−1^ recirculation; 4 h at 70 °C	Clean-water permeance 238 ± 9 L m^−2^ h^−1^ bar^−1^.	Dynamic removal 3.4 ± 0.3 mmol g^−1^ particles at 4 h; static removal 2.1 ± 0.1 mmol g^−1^ particles at 24 h; projected 215 g MMM (3.7 m^2^ outer area) for 400 mmol day^−1^.	Glucose and electrolytes were present in the model dialysate, but their recovery was NR; competing nucleophiles were not tested dynamically.	The 70 °C reaction condition and particle-mass normalization prevent direct translation to a 37 °C wearable cartridge.
Three-component DLHF: PSf/AC inner layer and PSf/PMMA outer layer [22]	Aqueous creatinine and urea; ultrafiltration–adsorption experiment; device-scale continuous runtime NR	Pure-water flux up to 25.2 L m^−2^ h^−1^.	Creatinine-plus-urea combined mean removal of 29.3%; separate urea value NR; reported urea uptake recovery of 81.2%.	Maximum creatinine adsorption capacity 86.2 mg g^−1^ at 9 wt% AC; reported creatinine uptake recovery of 98.8%; glucose/electrolyte data NR.	Finite-volume combined removal cannot establish urea-specific clearance, breakthrough, or wearable runtime.
Amine-MPS DLHF-MMM: PES/PEG inner layer and amine-functionalized MPS outer layer [18]	Actual patient spent dialysate; multisolute test; long-duration breakthrough/runtime NR	Water permeability 298.6 ± 3.2 mL m^−2^ h^−1^ mmHg^−1^.	Reported adsorption capacity 523.5 mg g^−1^; urea removal 74.4%.	Reported adsorption capacities: creatinine 28.1 and hippuric acid 3.1 mg g^−1^; removal: creatinine 56.0%, hippuric acid 16.1%, and spiked lysozyme 58.7%.	Patient-derived feed and multisolute measurements improve relevance; total module mass, repeated use, sterilization, and long-duration breakthrough remain unresolved.
Blood-side AC/PES/PVP mixed-matrix dialysis fiber [19]	Human plasma and endotoxin-contaminated dialysate; in vitro; not a spent-dialysate urea-regeneration module	Water permeance 11.4 L m^−2^ h^−1^ bar^−1^ for the mixed-matrix fiber; 19.5 L m^−2^ h^−1^ bar^−1^ for the PES/PVP control.	Urea was not the target; NR.	Combined 4 h plasma-toxin/endotoxin tests: endotoxin (415 ± 59) × 10^3^ EU m^−2^; hippuric acid 2267 ± 159 mg m^−2^; indoxyl sulfate 494 ± 96 mg m^−2^. Separate controls gave 375 ± 72 × 10^3^ EU m^−2^ without protein-bound toxins, and 2573 ± 175/420 ± 67 mg m^−2^ for hippuric acid/indoxyl sulfate without endotoxin.	The study projected approximately 0.15 m^2^ of functional membrane area for daily indoxyl-sulfate/hippuric-acid removal plus endotoxin control. This blood-side application is relevant to the primary blood–dialysate interface and dialysate safety, but is not directly comparable with secondary regeneration modules.

Table 2 separates water transport, urea performance, and co-solute or safety measurements because flux, permeance, recovery, rejection, batch removal, adsorption capacity, and reaction rate are different endpoints. Ranges refer to membranes or conditions within one study; values estimated from plots are marked approximate. NR means that a quantitative value was not identified in the source material used for this table, not that performance was zero. Background shading distinguishes the header and alternating rows only; it does not encode quantitative values or evidence levels. Abbreviations: AC, activated carbon; CA, cellulose acetate; CTA, cellulose triacetate; DLHF, dual-layer hollow fiber; EUs, endotoxin units; FO, forward osmosis; MMM, mixed-matrix membrane; MMMA, mixed-matrix membrane adsorber; MPS, mesoporous silica; NF, nanofiltration; NR, not reported quantitatively in the source material used; PEG, poly (ethylene glycol); PES, polyethersulfone; PMMA, poly (methyl methacrylate); PSf, polysulfone; PVP, polyvinylpyrrolidone; psi, pounds per square inch; RO, reverse osmosis; TMP, transmembrane pressure; wt%, weight percent; ZSM-5, Zeolite Socony Mobil-5; ZUF, the clinoptilolite product designation used in the source. NF3, NF4, NF90, NF270, dnf40, and dnf80 are membrane product names; mmHg means millimeters of mercury.

**Table 3:** Translational assessment of membrane-enabled regeneration architectures.

Architecture	Current Evidence	Principal Unresolved Risk	Potential Role	Readiness
Primary dialyzer plus urease-sorbent train	Historical REDY experience; in vitro, animal, and limited wearable-system precedent [3,24,30]	Ammonium breakthrough, CO_2_/gas, electrolyte drift, cartridge mass, flow stability	Reference architecture for low-volume regeneration and sensor development	Moderate (8/10)
Selective FO membrane plus photo/electrochemical sink	24 h coupled FO–oxidation bench operation with human spent dialysate and continuous urea input [15]	By-products, draw and water balance, electrode area, power, long-duration fouling	Protected catalytic side loop	Moderate (6/10)
Commercial NF fractionation	Multisolute transport mapped across pressure, temperature, and feed conditions [17]	Electrolyte/glucose redistribution and absence of an integrated urea sink	Fractionation before a downstream capture or conversion stage	Low (1/10)
Hollow-fiber mixed-matrix adsorption	Standalone hollow-fiber experiments; patient-fluid scoring anchor [18], with earlier model-dialysate work [21] as context.	Breakthrough, total module mass, filler release, cleaning, sterilization	Replaceable secondary regeneration cartridge	Low (2/10)
Blood-side mixed-matrix dialysis membrane	In vitro plasma toxin removal and endotoxin-barrier evidence [19]	Hemocompatibility and long-term patency in integrated use	Enhanced primary membrane and dialysate safety barrier	Low to moderate (3/10)

Scores are anchored to one representative implementation per row to avoid combining the best results from unrelated prototypes. In the order evidence/feed/duration/safety/integration, the anchors and scores are human urease-sorbent WAK [3], 2/2/2/1/1 = 8; FO–photoelectrochemical prototype [15], 1/1/2/1/1 = 6; commercial NF screening [17], 0/1/0/0/0 = 1; amine-silica hollow-fiber adsorption [18], 0/1/0/0/1 = 2; and blood-side mixed-matrix fiber [19], 0/1/0/1/1 = 3. Unreported evidence receives 0 rather than an assumed favorable score. The risks listed identify unresolved functions; priority is given to return-stream toxicity or blood exposure, then loss of clearance or fluid/electrolyte control, and then engineering burdens. This proposed rubric is not clinically validated and does not estimate regulatory approval or time to market. The blood-side fiber is included as a primary-membrane comparator, not as a secondary urea-regeneration module. FO, forward osmosis; NF, nanofiltration; REDY, Recirculating Dialysis; WAK, wearable artificial kidney. Background shading distinguishes the header and alternating rows only; it does not encode quantitative values or evidence levels.

## Data Availability

No new experimental data were generated. The numerical comparisons and evidence assessments are based on the publications cited in this review.
